# Activation of PPARα Ameliorates Cardiac Fibrosis in Dsg2-Deficient Arrhythmogenic Cardiomyopathy

**DOI:** 10.3390/cells11203184

**Published:** 2022-10-11

**Authors:** Zirui Qiu, Yawen Zhao, Tian Tao, Wenying Guo, Ruonan Liu, Jingmin Huang, Geyang Xu

**Affiliations:** 1 Department of Physiology, School of Medicine, Jinan University, 601 Huangpu Avenue West, Tianhe District, Guangzhou 510632, China; 2 Center for Clinical Epidemiology and Methodology (CCEM), Guangdong Second Provincial General Hospital, Guangzhou 510317, China

**Keywords:** arrhythmogenic cardiomyopathy, desmoglein-2, cardiac fibrosis, PPARα, fenofibrate, STAT3

## Abstract

**Highlights:**

- Cardiac-specific Dsg2 deletion induces excessive cardiac fibrosis in mice.
- Fenofibrate alleviates cardiac fibrosis in CS-Dsg2^−/−^ mice.
- Cardiac-specific activation of PPARα ameliorates cardiac fibrosis in CS-Dsg2^−/−^ mice.
- The inhibitory effect of PPARα on cardiac fibrosis is mediated by STAT3 and TGF-β /SMAD3 signaling.
- PPARα is a promising target for the intervention of ACM by ameliorating cardiac fibrosis.

**Abstract:**

Background: Arrhythmogenic cardiomyopathy (ACM) is a genetic heart muscle disease characterized by progressive fibro-fatty replacement of cardiac myocytes. Up to now, the existing therapeutic modalities for ACM are mostly palliative. About 50% of ACM is caused by mutations in genes encoding desmosomal proteins including Desmoglein-2 (Dsg2). In the current study, the cardiac fibrosis of ACM and its underlying mechanism were investigated by using a cardiac-specific knockout of Dsg2 mouse model. Methods: Cardiac-specific *Dsg2* knockout (CS-Dsg2^−/−^) mice and wild-type (WT) mice were respectively used as the animal model of ACM and controls. The myocardial collagen volume fraction was determined by histological analysis. The expression levels of fibrotic markers such as α-SMA and Collagen I as well as signal transducers such as STAT3, SMAD3, and PPARα were measured by Western blot and quantitative real-time PCR. Results: Increased cardiac fibrosis was observed in CS-Dsg2^−/−^ mice according to Masson staining. PPARα deficiency and hyperactivation of STAT3 and SMAD3 were observed in the myocardium of CS-Dsg2^−/−^ mice. The biomarkers of fibrosis such as α-SMA and Collagen I were upregulated after gene silencing of Dsg2 in HL-1 cells. Furthermore, STAT3 gene silencing by Stat3 siRNA inhibited the expression of fibrotic markers. The activation of PPARα by fenofibrate or AAV9-Pparα improved the cardiac fibrosis and decreased the phosphorylation of STAT3, SMAD3, and AKT in CS-Dsg2^−/−^ mice. Conclusions: Activation of PPARα alleviates the cardiac fibrosis in ACM.

## 1. Introduction

Arrhythmogenic cardiomyopathy (ACM) is a fatal heart disease characterized by cardiac dysfunction, heart failure, and life-threatening ventricular arrhythmias [1,2]. The population prevalence of ACM has been estimated between 1:1000 and 1:5000 [2]. Studies have shown that ACM causes 10% to 15% of sudden cardiac death (SCD) cases, especially among young people and athletes [2]. Pathological features of ACM include loss of myocytes and progressive fibro-fatty replacement. These pathological features tend to occur in the right ventricle (RV), with left ventricular (LV) or bilateral ventricular involvement [2,3]. Previous studies found that one or more mutations in genes encoding desmosomal proteins led to about 50% of ACM cases [4], including desmoglein2 (DSG2) [5], desmocollin2 [6], plakoglobin [7], desmoplakin [8], and plakophilin-2 [9]. DSG2 is a major cadherin of the cardiac desmosome; it was reported that mutations in the Dsg2 gene are associated with severe lethal heart muscle diseases such as ACM [10]. Till now, the main purpose of existing treatment for ACM is to prevent SCD [11].

Necrotic and apoptotic cardiomyocytes are replaced by fibrosis during the ACM disease’s progress [12]. Although cardiac fibrosis plays a critical role in enhancing cardiac structural stability, it results in cardiac structural remodeling and impaired cardiac function, finally increasing the risk of potentially lethal cardiac arrhythmias [13]. Thus, improvement of cardiac fibrosis might be beneficial to avoid further deterioration of the ACM. The signal transducer and activator of transcription 3 (STAT3) is hyperactivated in fibrotic diseases and STAT3 inhibitors are currently used in the treatments of fibrotic diseases, especially in cardiac fibrosis [14]. Transforming growth factor-β (TGF-β) is a central mediator in hypertrophic and fibrotic of the heart. Canonical and non-canonical pathways for TGF-β-induced fibrosis in the heart are known [15]. Furthermore, interaction of Stat3 and TGF-β/Smad3 signaling is regarded as playing a critical role in cardiac fibrotic processes [16]. Recent studies illustrated the antagonistic effects and bidirectional regulation between STATs and peroxisome proliferator-activated receptors (PPARs) and suggested a potential cross-talk between STAT and PPAR pathways [17,18,19]. PPARs are the nuclear receptor superfamily of ligand-activated transcription factors. As the predominant PPAR isoform in the heart, peroxisome proliferator-activated receptor α (PPARα) modulates cardiac metabolism substrate conversion in cardiac hypertrophy, cardiac hypoxia, and diabetic heart [20]. PPARα gene deletion contributes to cardiac hypertrophy and deterioration of cardiac function [21]. Previous studies illustrated that PPARα activation alleviated cardiac fibrosis and reversed cardiac dysfunction [22] and PPARα could inhibit the TGF-β-induced profibrotic pathway in cardiac fibrosis [23,24]. Fenofibrate alleviated myocardial inflammation and collagen deposition in Ang II-infused rats [25]. Recently, we reported that activation of PPARα reduced the cardiac lipid accumulation and restored cardiac function in ACM mice [26]. Although PPARα plays a critical role in lipid accumulation in ACM, the effects of PPARα on cardiac fibrosis in ACM is still unclear. We hypothesized that the PPARα-STAT3/SMAD pathway is critical to cardiac fibrosis in ACM mice. In our current study, we found that PPARα was downregulated in the hearts of cardiac-specific Dsg2 knockout mice; restoring the activity of PPARα by using fenofibrate (a PPARα agonist) or AAV9-Pparα improved cardiac fibrosis via the PPARα-STAT3/SMAD pathway in cardiac-specific Dsg2 deletion mice. Our findings suggest that PPARα is a potential therapeutic target of cardiac fibrosis in ACM. 

## 2. Materials and Methods

### 2.1. Materials

Fenofibrate was purchased from Sigma-Aldrich (St. Louis, MO, USA). Rabbit anti-Phospho-stat3 (Tyr705), rabbit anti-Phospho-SMAD3 (Ser423/425), rabbit anti-SMAD, rabbit anti-Phospho-AKT (Ser473), rabbit anti-AKT, rabbit anti-α-SMA, rabbit anti-Collagen I antibodies, mouse anti-stat3, and mouse monoclonal anti-β-actin were purchased from Cell Signaling Technology (Beverly, MA, USA). Rabbit anti-DSG2, rabbit anti-PPARα, rabbit anti-GAPDH antibodies were from Abcam Inc. (Cambridge, MA, USA). 

### 2.2. Animals and Treatments

Cardiac-specific dsg2 gene knockout (CS-Dsg2^−/−^) on C57-based genetic backgrounds were successfully constructed by mating DSG2 flox with CKMM cre [26]. Mice were housed in standard plastic rodent cages and maintained in a regulated environment (24 °C, 12 h light and 12 h dark cycles with lights on at 7:00 a.m.). All mice used in this study were 8–12 weeks old.

In order to activate PPARα in vivo, PPARα agonist fenofibrate (150 mg/kg body weight) was administrated by daily oral gavage for 28 days. To overexpress PPARα in the heart, male CS-Dsg2^−/−^ mice were tail-vein infused with adeno-associated virus carrying PPAR*α* (AAV9-cTnT-*Pparα*, 5 × 10^11^ vg per mouse). Adeno-associated virus carrying GFP (AAV9-cTnT-GFP, 5 × 10^11^ vg per mouse) was used as control. The mice were sacrificed 28 days after AAV injections [26].

### 2.3. Histological Analysis

Hearts were harvested from mice, fixed overnight in 4% paraformaldehyde, embedded in paraffin, and then, sectioned serially. Masson’s trichrome staining was performed to evaluate collagen deposition using a kit following manufacturer’s instructions (G1006-20 ML, Servicebio, Wuhan, China). The collagen volume fraction (CVF) was determined by Image J software as an index of cardiac fibrosis. The ratio of myocardial collagen area to the total myocardial area was used to calculate the collagen volume fraction.

### 2.4. Cell Culture and Treatment

The murine atrial cardiac myocyte cell line HL-1 was maintained in 10% fetal bovine serum (FBS) in Dulbecco’s modified Eagle’s medium at 37 °C in an atmosphere of 5% CO_2_. For transient transfection, cells were plated at optimal densities and grown for 24 h. Cells were then transfected with Dsg2 siRNA (MBS828119, MyBioSource, San Diego, CA, USA) or Stat3 siRNA (6354, Cell Signaling Technology) using lipofectamine reagent according to the manufacturer’s instructions. 

### 2.5. Western Blot Analysis

The tissues and cells were homogenized in the lysis buffer. After protein quantification, 40 μg of protein was loaded onto SDS-PAGE gels. Then, protein extracts were electrophoresed, blotted, and then, incubated with primary antibodies. The antibodies were detected using 1:10,000 horseradish peroxidase-conjugated donkey anti-rabbit IgG and donkey anti-mouse IgG (Jackson ImmunoResearch, West Grove, PA, USA). Western blotting luminol reagent was used to visualize bands. The band intensities were quantitated by Image J software. 

### 2.6. RNA Extraction, Quantitative Real-Time PCR

For gene expression analysis, RNA was isolated from mouse tissues and cells by using Trizol (Takara, Kusatsu, Shiga) and reverse-transcribed into cDNAs using the first-strand synthesis system for RT-PCR kit (Takara). SYBR green-based real-time PCR was performed using the Bio-Rad IQ5 PCR system (Bio-Rad, Foster City, CA, USA). Sequences for the primer pairs used in this study are shown in Table 1.

### 2.7. Statistical Analysis

Data are expressed as mean ± SEM. Statistical significance was analyzed with a student’s t-test. Differences were considered statistically significant with *p* values < 0.05.

## 3. Results 

### 3.1. Cardiac-Specific Dsg2 Gene Deletion Provokes Cardiac Fibrosis

**Figure 1:**
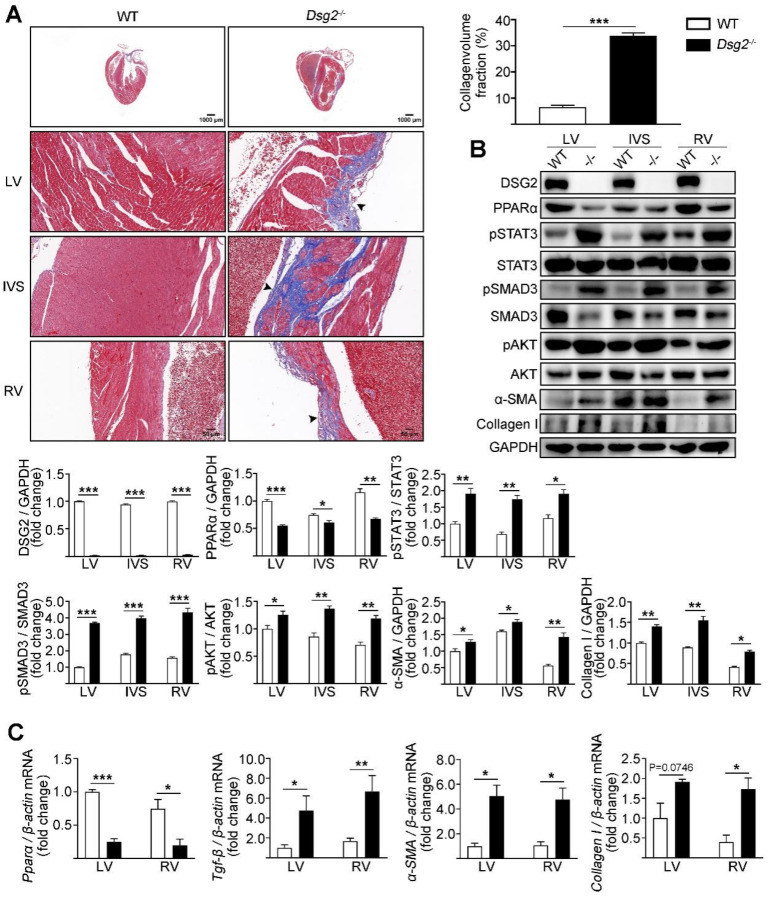
Cardiac-specific Dsg2 knockout induced cardiac fibrosis. (**A**) Masson staining of heart sections in WT and CS-Dsg2^−/−^ (−/−) mice. Arrow shows cardiac fibrosis. Collagen volume fraction in the hearts of WT and CS-Dsg2^−/−^ mice was assessed. (**B**) Representative Western blots from mouse left ventricular (LV), interventricular septum (IVS), and right ventricle (RV). DSG2, PPARα, pSTAT3, pSMAD3, pAKT, α-SMA, and Collagen I were detected using specific antibodies. STAT3, SMAD3, AKT, and GAPDH were used as loading controls. (**C**) Results of quantitative PCR analysis of PPARα, TGF-β, α-SMA, and Collagen I mRNA levels in mouse LV and RV are expressed as fold change of control using β-actin as loading control. Results are expressed as mean values ± SEM. n = 6. * *p* < 0.05, ** *p* < 0.01, *** *p* < 0.001 vs. WT.

The ACM mouse model was generated by crossing Dsg2^fl-neo/+^ mice with Ckmm-Cre mice which resulted in cardiac-specific Dsg2 deletion (CS-Dsg2^−/−^). Increased cardiac fibrosis was observed in CS-Dsg2^−/−^ mice according to Masson staining (Figure 1A). Several studies indicated that the activation of STAT3 contributes to cardiac fibrosis [27,28,29]. In our study, increased phosphorylation levels of STAT3 at Tyr705, SMAD3 at Ser423/425, and AKT at Ser473, and decreased expression levels of PPARα were observed in the LV, interventricular septum (IVS), and RV of CS-Dsg2^−/−^ mice (Figure 1B). We next investigated the expression of TGF-β, α-smooth muscle actin (α-SMA), and collagen type I (Collagen I) in CS-Dsg2^−/−^ mice. Expression levels of TGF-β, α-SMA, and Collagen I in LV, IVS, and RV of CS-Dsg2^−/−^ mice were higher than that of littermate controls (Figure 1B,C).

**Figure 2:**
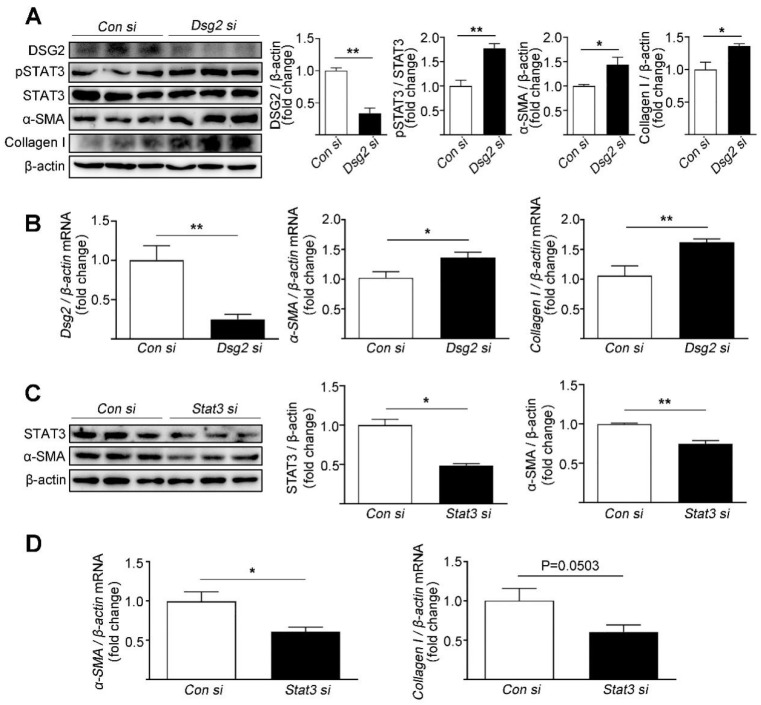
Effects of Dsg2 siRNA and Stat3 siRNA on the expression levels of fibrotic markers in HL-1 cells. (**A**,**B**) HL-1 cells were transfected with control siRNA or Dsg2 siRNA. (**A**) Representative Western blots for DSG2, pSTAT3, α-SMA, and Collagen I were detected using specific antibodies. STAT3 and β-actin were used as loading controls. (**B**) Results of quantitative PCR analysis of Dsg2, α-SMA, and Collagen I mRNA levels in HL-1 cells treated with control or Dsg2 siRNA are expressed as fold change of control using β-actin as loading control. Results are expressed as mean values ± SEM. n = 3. * *p* < 0.05, ** *p* < 0.01 vs. control. (**C**,**D**) HL-1 cells were transfected with control siRNA or Stat3 siRNA. (**C**) Representative Western blots for STAT3 and α-SMA were detected using specific antibodies. β-actin were used as loading controls. (**D**) Results of quantitative PCR analysis of α-SMA and Collagen I mRNA levels in HL-1 cells treated with control or Stat3 siRNA are expressed as fold change of control using β-actin as loading control. Results are expressed as mean values ± SEM. n = 3. * *p* < 0.05, ** *p* < 0.01, vs. control.

The effects of Dsg2 on STAT3 activity and fibrosis were assessed in the cardiac myocyte cell line HL-1. To silence the expression of Dsg2 and Stat3, HL-1 cells were transfected with Dsg2 siRNA and Stat3 siRNA. The knockdown efficiency of Dsg2 siRNA was 66% and 51% for Stat3 siRNA. Consistent to the in vivo study, knockdown of Dsg2 in the HL-1 cells led to an increase in the phosphorylation of STAT3 (Tyr705) and the expression levels of α-SMA and Collagen I (Figure 2A,B). Furthermore, knockdown of Stat3 in the HL-1 cells decreased the expression levels of α-SMA and Collagen I (Figure 2C,D).

### 3.2. Fenofibrate Alleviated Cardiac Fibrosis in CS-Dsg2^−/−^ Mice

**Figure 3:**
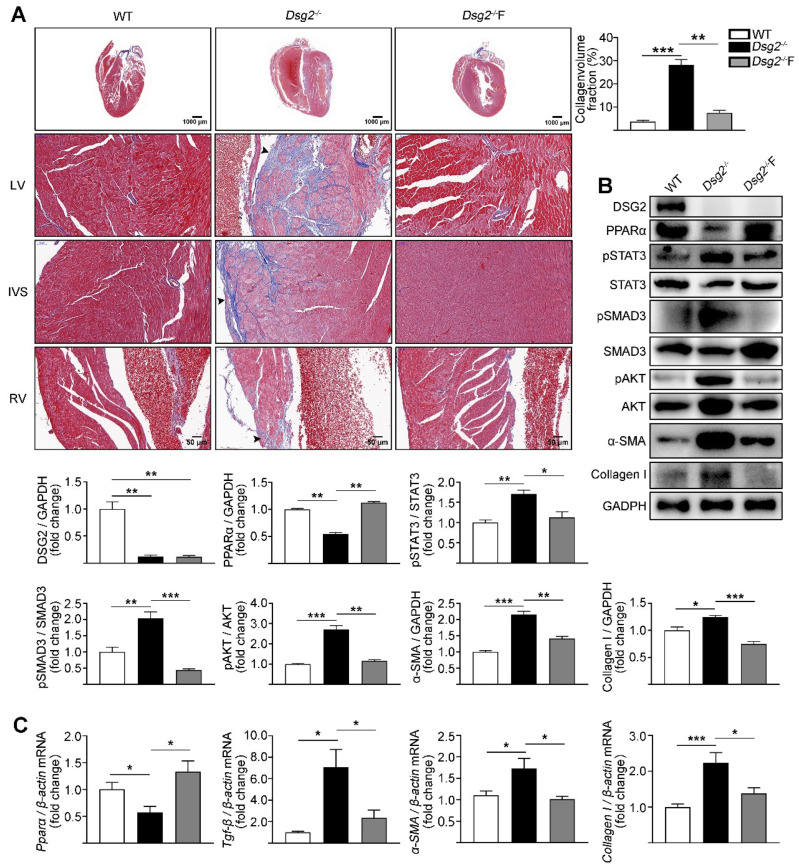
Fenofibrate alleviated cardiac fibrosis in CS-Dsg2^−/−^ mice. (**A**) Masson staining of heart sections in WT, CS-Dsg2^−/−^ mice, and CS-Dsg2^−/−^ mice treated with fenofibrate (Dsg2^−/−^F). Collagen volume fraction in the hearts of WT, CS-Dsg2^−/−^, and Dsg2^−/−^F mice were assessed. (**B**) Representative Western blots from ventricles of WT, CS-Dsg2^−/−^, and Dsg2^−/−^F mice. DSG2, PPARα, pSTAT3, pSMAD3, pAKT, α-SMA, and Collagen I were detected using specific antibodies. STAT3, SMAD3, AKT, and GAPDH were used as loading controls. (**C**) Results of quantitative PCR analysis of PPARα, TGF-β, α-SMA, and Collagen I mRNA levels in mouse ventricles are expressed as fold change of control using β-actin as loading control. Results are expressed as mean values ± SEM. n = 6. * *p* < 0.05, ** *p* < 0.01, *** *p* < 0.001 vs. control.

Fenofibrate, a PPARα agonist, affords myocardial protection apart from its lipid lowering effects [30]. We next assessed the effect of fenofibrate on cardiac fibrosis in CS-Dsg2^−/−^ mice. Interestingly, a significant improvement in cardiac fibrosis was observed in CS-Dsg2^−/−^ mice after being treated with fenofibrate (150 mg/kg/day, for 4 weeks) (Figure 3A). Fenofibrate decreased the phosphorylation levels of STAT3, SMAD3, and AKT as well as the expression levels of TGF-β, α-SMA, and Collagen I in CS-Dsg2^−/−^ mice (Figure 3B,C).

### 3.3. Cardiac-Specific Activation of PPARα Alleviated Cardiac Fibrosis in CS-Dsg2^−/−^ Mice

**Figure 4:**
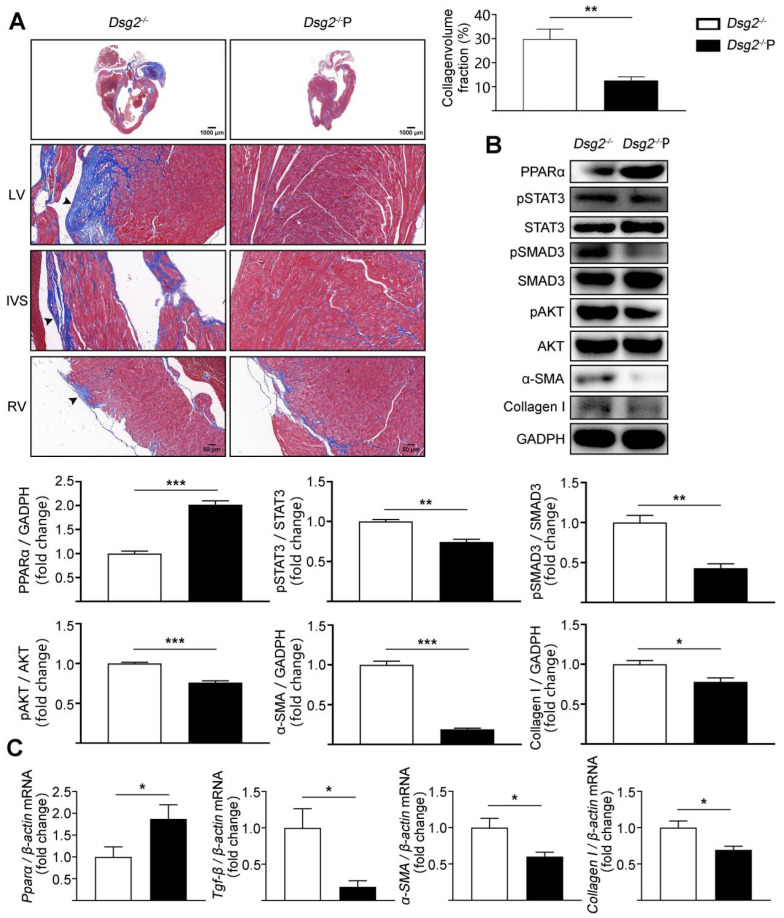
AAV9-Pparα alleviated cardiac fibrosis in CS-Dsg2^−/−^ mice. (**A**) Masson staining of heart sections in CS-Dsg2^−/−^ mice and CS-Dsg2^−/−^ mice received AAV9-Pparα (Dsg2^−/−^P). Collagen volume fraction in the hearts of CS-Dsg2^−/−^ and Dsg2^−/−^P mice were assessed. (**B**) Representative Western blots from ventricles of CS-Dsg2^−/−^ and Dsg2^−/−^P mice. PPARα, pSTAT3, pSMAD3, pAKT, α-SMA, and Collagen I were detected using specific antibodies. STAT3, SMAD3, AKT, and GAPDH were used as loading controls. (**C**) Results of quantitative PCR analysis of PPARα, TGF-β, α-SMA, and Collagen I mRNA levels in mouse ventricles are expressed as fold change of control using β-actin as loading control. Results are expressed as mean values ± SEM. n = 6. * *p* < 0.05, ** *p* < 0.01, *** *p* < 0.001 vs. CS-Dsg2^−/−^.

To further confirm that PPARα activation in the heart could improve cardiac fibrosis in Dsg2^−/−^ mice, cardiac-specific activation of PPARα was performed by tail-vein infusion of AAV9. AAV9-cTnT promoter-Pparα significantly reduced cardiac fibrosis in CS-Dsg2^−/−^ mice (Figure 4A). Simultaneously, the levels of phosphorylated STAT3, phosphorylated SMAD3, phosphorylated AKT, and the expression levels of TGF-β, α-SMA, and Collagen I were decreased after cardiac-specific activation of PPARα (Figure 4B,C).

## 4. Discussion

ACM is characterized by progressive replacement of cardiomyocytes by fibro-fatty tissue, cardiac dysfunction, ventricular arrhythmias, and heart failure. Mutation of desmoglein-2 (Dsg2) is one of the major causes of ACM and has been shown to lead to a loss of adhesive function [31]. Dsg2 mutation carriers display more severe heart muscle disease, which is associated with biventricular involvement and rapid evolution to end-stage heart failure [32]. In our previous study, we generated an ACM mouse model by cardiac-specific knockout of the DSG2 gene and discovered that downregulation of PPARα contributed to the impairment of fatty acid oxidation and, thus, to lipid accumulation in the DSG2 deletion-induced ACM [26]. However, whether downregulation of PPARα also contributes to the fibrosis in ACM was unsolved. In the present study, we uncovered a previously unrecognized role of PPARα in cardiac fibrosis in Dsg2-deficient ACM mice. Cardiac-specific Dsg2 knockout contributed to a severe cardiac fibrosis. Decreased expression of PPARα and the increased phosphorylation of STAT3 and SMAD3 were observed in this model. Moreover, activation of PPARα, either by fenofibrate or AAV9-Pparα, decreased the activity of STAT3 and SMAD3 and improved cardiac fibrosis in Dsg2 deletion-induced ACM.

Cardiac fibrosis is defined as excessive deposition of extracellular matrix (ECM) proteins by cardiac fibroblasts (CFs). CFs are transformed into myofibroblasts when they respond to stress and pathological stimuli [33]. Myocardial fibrosis reduces tissue compliance and accelerates the progression to heart failure [34]. In our study, histological analysis showed excessive deposition of collagen in the hearts of CS-Dsg2^−/−^ mice when compared to WT mice. Furthermore, fibrotic markers such as TGF-β, α-SMA, and Collagen I were activated after cardiac-specific Dsg2 deletion. Cardiac fibrosis has been implicated in the progression of ACM. Increased cardiac fibrosis has been associated with altered cardiac conduction, resulting in conduction slowing, blockage, and re-entry [35]. Recent evidence indicates that the fibrosis state preceded the development of cardiac dysfunction in cardiomyopathies [36]. Thus, identification of druggable targets that can alleviate cardiac fibrosis might be beneficial to the treatment of ACM.

As a ligand-activated transcription factor which is highly expressed in cardiomyocytes, the role of PPARα in the heart is complex and vital. PPARα involvement in the regulation of inflammation [37], hypertrophy [38], energy metabolism [39], ischemia/reperfusion injury [40], and cardiac fibrosis [25] in hearts has been established in recent studies. Fenofibrate is a member of the fibrate family of PPARα receptor agonists and has regulating efficacy of inflammation and extracellular matrix remodeling of the heart [25,41]. As a PPARα agonist, fenofibrate has been widely used for hyperlipidemia in clinics and can also promote fatty acid oxidation in the mitochondria and improve myocardial energy metabolism [42]. Fenofibrate alleviated myocardial inflammation and fibrosis in diabetic mice via PPARα receptor [43]. Our previous study showed that PPARα was downregulated in the heart of the Dsg2 deletion ACM model and reactivation of PPARα significantly alleviated the lipid accumulation and improved cardiac function in CS-Dsg2^−/−^ mice [26]. In the current study, we demonstrated that downregulation of PPARα also contributed to the cardiac fibrosis in the Dsg2 deletion-induced ACM model. Moreover, reactivation of PPARα either by tail-vein injection of AAV9-Pparα or oral treatment of fenofibrate improved the cardiac fibrosis in CS-Dsg2^−/−^ mice. Our results suggested that PPARα is a promising therapeutic target for ACM intervention which not only alleviates lipid accumulation but also improves cardiac fibrosis.

Although cardiac fibrosis is one of the pathological characteristics of ACM, the mechanism of how mutations of desmosomal proteins lead to fibrosis is elusive. TGF-β is a core mediator in cardiac fibrosis. Canonical (SMAD-dependent) and non-canonical (SMAD-independent) pathways for TGF-β-induced fibrosis in the heart are documented [44]. In the canonical pathway, TGF-β activates SMAD2/3 signaling, which in turn regulates the expressions of collagen and α-SMA in myofibroblasts [45]. Non-canonical pathways involve STAT, MAPK, and PI3K pathways [46,47,48]. Our study showed that cardiac-specific Dsg2 deletion led to enhanced phosphorylation of SMAD3, STAT3, and AKT, suggesting that both canonical and non-canonical TGF-β pathways are activated in Dsg2 deletion-induced ACM. Among these pathways, STAT3 is reported to be critical to cardiac fibrosis and hypertrophy and activated in the hearts of mouse models of cardiac hypertrophy and heart failure [49]. Several studies have demonstrated that STAT3 maintains ECM homeostasis by regulating collagen synthesis and secretion in CFs [50]. Continuous STAT3 activation (tyrosine 705 residue phosphorylation) was regarded as a poor indicator in cardiac hypertrophy and heart failure [51]. Our study showed that knockdown of Dsg2 by Dsg2 siRNA induced the activation of fibrotic markers and STAT3 in HL-1 cells, while Stat3 siRNA did the reverse. These results suggested that activation of STAT3 contributes to cardiac fibrosis in cardiac-specific Dsg2 deletion mice. Furthermore, reactivation of PPARα either by AAV9-PPARα or fenofibrate decreased the phosphorylation of SMAD3, STAT3, and AKT in the Dsg2 deletion-induced ACM model, implying that PPARα modulated these pathways and deficiency in PPARα contributed to the activation of them and, thus, to cardiac fibrosis. Although the mechanistic link between PPARα and the STAT3 and TGF-β /SMAD3 pathways remains unclear, potential cross-talk between PPARα and STAT3 and TGF-β /SMAD3 pathways were reported in recent studies [17]. Chang H et al. reported that activation of PPARα ameliorates autoimmune myocarditis by suppressing Th17 cell differentiation through reducing phosphorylated STAT3 [52]. Gervois et al. demonstrated that fenofibrate treatment decreased the phosphorylation of STAT3 in livers [53]. Bansal T et al. reported that activation of PPARα improves cardiac fibrosis by inhibiting non-canonical TGF-β signaling [24]. Sekiguchi K et al. demonstrated that TGF-β signaling pathways directly inhibit PPARα activity in cardiac myocytes [54]. These studies suggest a role of PPARα in modulating STAT3 and TGF-β /SMAD3 pathways.

Current treatments for ACM lack effective treatment to improve or reverse cardiac fibrosis. In the present study, we established that activation of PPARα by fenofibrate or AAV9-Pparα improved the cardiac fibrosis in Dsg2 deletion-induced ACM. At the same time, activation of PPARα provided a cardioprotective effect through reducing the phosphorylation of STAT3 and SMAD3. These results indicated that the inhibitory effect of PPARα on cardiac fibrosis is mediated by a downregulation of STAT3 and TGF-β /SMAD3, and PPARα may be a significant target of ACM treatment. PPARα agonist fenofibrate may be a potential drug against cardiac fibrosis in ACM. In conclusion, our study generated an ACM model by cardiac-specific Dsg2 knockout and suggested that activation of PPARα ameliorates the excessive cardiac fibrosis in ACM.

## Figures and Tables

**Table 1:** List and sequences of primers used in RT-PCR experiments.

	Upstream Primer (5′-3′)	Downstream Primer (5′-3′)	Accession Number(s)
α-SMA	CCCTGAAGAGCATCCGACAC	TGCTGTTATAGGTGGTTTCGTG	NM_007392.3
Collagen I	TGTTCAGCTTTGTGGACCTC	GGACCCTTAGGCCATTGTGT	NM_007742.4
Dsg2	CGCACCAGGAAAGTACCAG	CCACAGTGGCATATCAACAGC	NM_007883.3
PPARα	AGAGCCCCATCTGTCCTCTC	ACTGGTAGTCTGCAAAACCAAA	XM_006520624.3
TGF-β	AGCCCTGGATACCAACTATTGCTTCAGCTCCACAG	AGGGGCGGGGCGGGGCGGGGCTTCAGCTGC	NM_011577.2
β-actin	CCACAGCTGAGAGGGAAATC	AAGGAAGGCTGGAAAAGAGC	NM_007393.5

## Data Availability

All data relevant to the study are included in the article or uploaded as supplemental information.

## Supplementary Materials

The following supporting information can be downloaded at: https://www.mdpi.com/article/10.3390/cells11203184/s1, Figure S1: The expression of PPARα in the skeletal muscle of cardiac specific DSG2 null mice received with AAV9-cTnT-GFP (DSG2^−/−^) or AAV9-cTnT-Pparα (DSG2^−/−^ P).

## Abbreviations

ACM	arrhythmogenic cardiomyopathy
CFs	cardiac fibroblasts
Collagen I	collagen type I
DSG2	Desmoglein-2
LV	left ventricular
PPARα	peroxisome proliferator-activated receptor α
RV	right ventricle
STAT3	signal transducer and activator of transcription 3
SMAD	mothers against decapentaplegic homolog 3
SCD	sudden cardiac death
TGF-β	transforming growth factor–β
α-SMA	alpha-smooth muscle actin
