# Supplementary material for: Activation of PPARα Ameliorates Cardiac Fibrosis in Dsg2-Deficient Arrhythmogenic Cardiomyopathy
Source: Cells. 2022 Oct 11;11(20):3184. doi: 10.3390/cells11203184 (PMC9601208; doi:10.3390/cells11203184)
Supplement: Supplementary file 1 [file cells-11-03184-s001.zip › cells-1893336-supplementary.pdf]

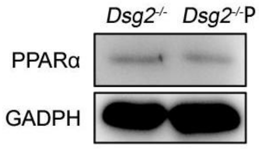

Figure S1. The expression of PPARα in the skeletal muscle of cardiac specific DSG2 null mice received with AAV9-cTnT-GFP ($DSG2^{-/-}$) or AAV9-cTnT-Pparα ($DSG2^{-/-}$ P)
